# Bilateral posterior scleritis after sequential bilateral cataract surgery: a case report

**DOI:** 10.1186/s12886-022-02537-1

**Published:** 2022-07-26

**Authors:** Chae-Min Hong, Min-Ho Shin, Seong-Jae Kim, Seong-Wook Seo, Inyoung Chung, Woong-Sun Yoo

**Affiliations:** 1 Department of Ophthalmology, College of Medicine, Gyeongsang National University, Gangnam-ro 79, Gyeongnam Jinju, South Korea; 2 Department of Ophthalmology, Gyeongsang National University Hospital, Gangnam-ro 79, Gyeongnam Jinju, South Korea; 3 Department of Ophthalmology, College of Medicine, Chosun University, Gwangju, South Korea; 4 Institute of Health Sciences, Gyeongsang National University, Gangnam-ro 79, Gyeongnam Jinju, South Korea

**Keywords:** Cataract surgery, Posterior scleritis, Steroids

## Abstract

**Background:**

Posterior scleritis is a rare, inflammatory ophthalmic disease, leading to severe visual impairment if untreated. Posterior scleritis occurring after surgery, unrelated to systemic inflammatory diseases, is even rarer. This report discusses a case of bilateral posterior scleritis, after cataract surgery in both the eyes, treated with high-dose steroids.

**Case presentation:**

A 55-year-old man, who had undergone bilateral sequential cataract surgery one week before, presented with sudden loss of vision and ocular pain in both eyes. The patient had no systemic diseases or neurological symptoms. Serous retinal detachment of the macula with optic disc swelling was observed on fundus examination in both the eyes, and bilateral thickening of choroid and sclera was seen in ultrasonography. Under diagnosis of bilateral posterior scleritis due to the increased signal of sclera in both the eyes on magnetic resonance imaging, high-dose steroid therapy was performed. After treatment, improvement in visual acuity and retinal detachment were observed, and thereafter, it has been maintained without relapse.

**Conclusions:**

With high-dose steroid therapy, we successfully treated a rare case of bilateral posterior scleritis following cataract surgery in both eyes. To our knowledge, this is the first report on posterior scleritis occurring after surgery, unrelated to systemic inflammatory diseases.

## Background

Posterior scleritis is the rarest among scleritis, an inflammatory ophthalmic disease, and can cause severe visual impairment if not treated adequately. The disease is commonly accompanied by serous retinal detachment and the invasion of the optic nerve and must be differentiated from infectious, neoplastic, vascular, and inflammatory diseases such as infectious neuroretinitis, sarcoidosis, choroidal tumors, Behcet’s disease and Vogt–Koyanagi–Harada syndrome [1]. It reportedly occurs mainly in women aged 30–60 years and is monocular in 85% of the cases. The diagnosis is difficult, especially if the condition occurs bilaterally, and differentiation from other inflammatory eye diseases is critical [2].

The causative factors of posterior scleritis vary, and include idiopathic, infectious, and traumatic causes; among which, non-infectious posterior scleritis is associated with systemic conditions such as rheumatic diseases in about 10% of the cases [3]. However, there have been no reports of posterior scleritis occurring after surgery, such as for cataract, not associated with a systemic inflammatory disease.

Herein, we report a case of bilateral posterior scleritis that occurred after cataract surgery in a healthy adult, which was successfully treated with high-dose systemic steroids.

## Case presentation

**Fig. 1:**
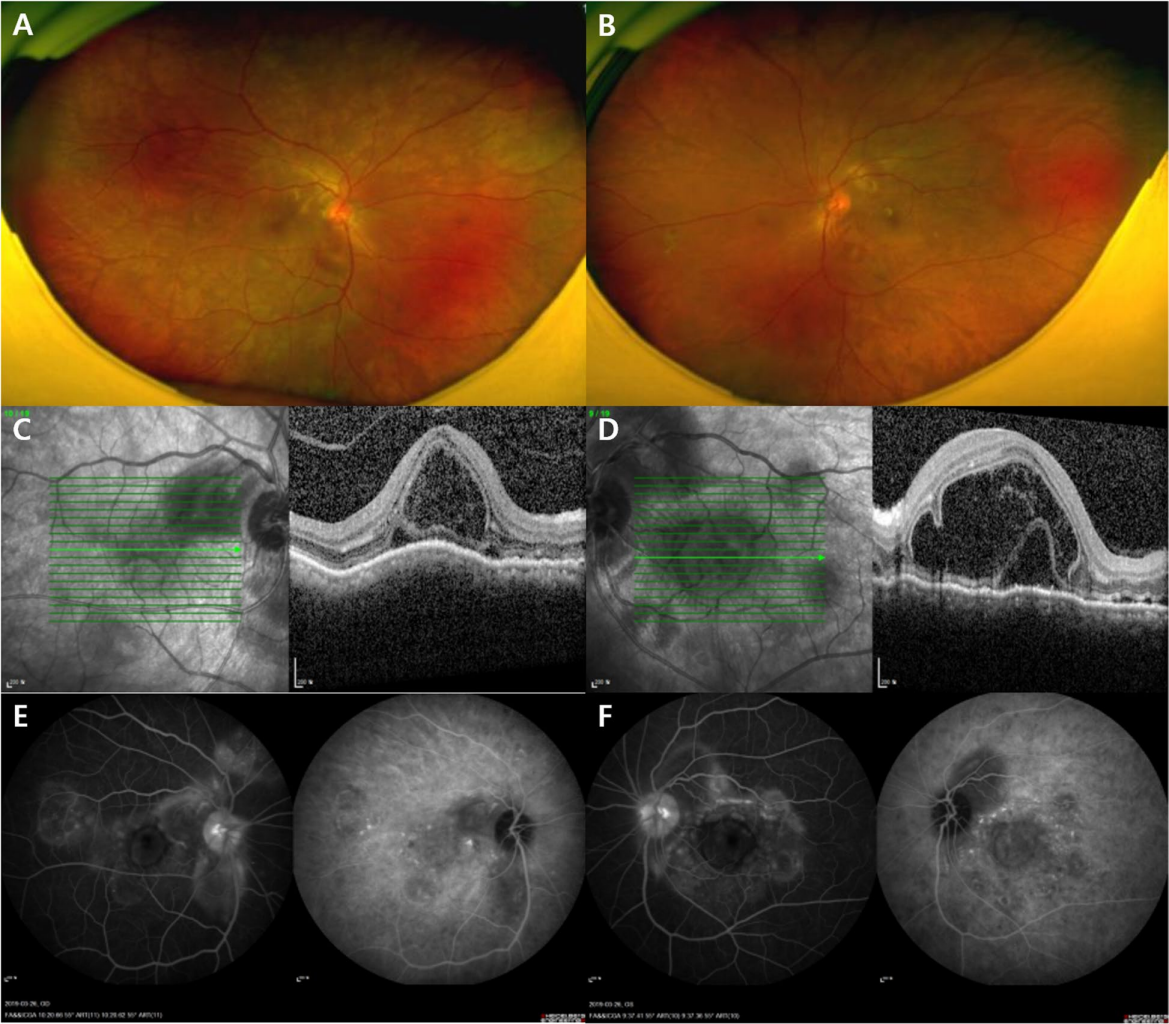
Fundus photography, optical coherence tomography (OCT), fluorescein angiography (FA), and indocyanine green angiography (ICGA) of the patient at initial visit. Fundus photography showed disk swelling with serous retinal detachment in the right (**a**) and left (**b**) eyes. OCT revealed serous retinal detachment with thickened choroid in the right (**c**) and left (**d**) eyes. FA and ICGA showed multiple leakages and staining lesions in the late phase in the right (**e**) and left (**f**) eyes

**Fig. 2:**
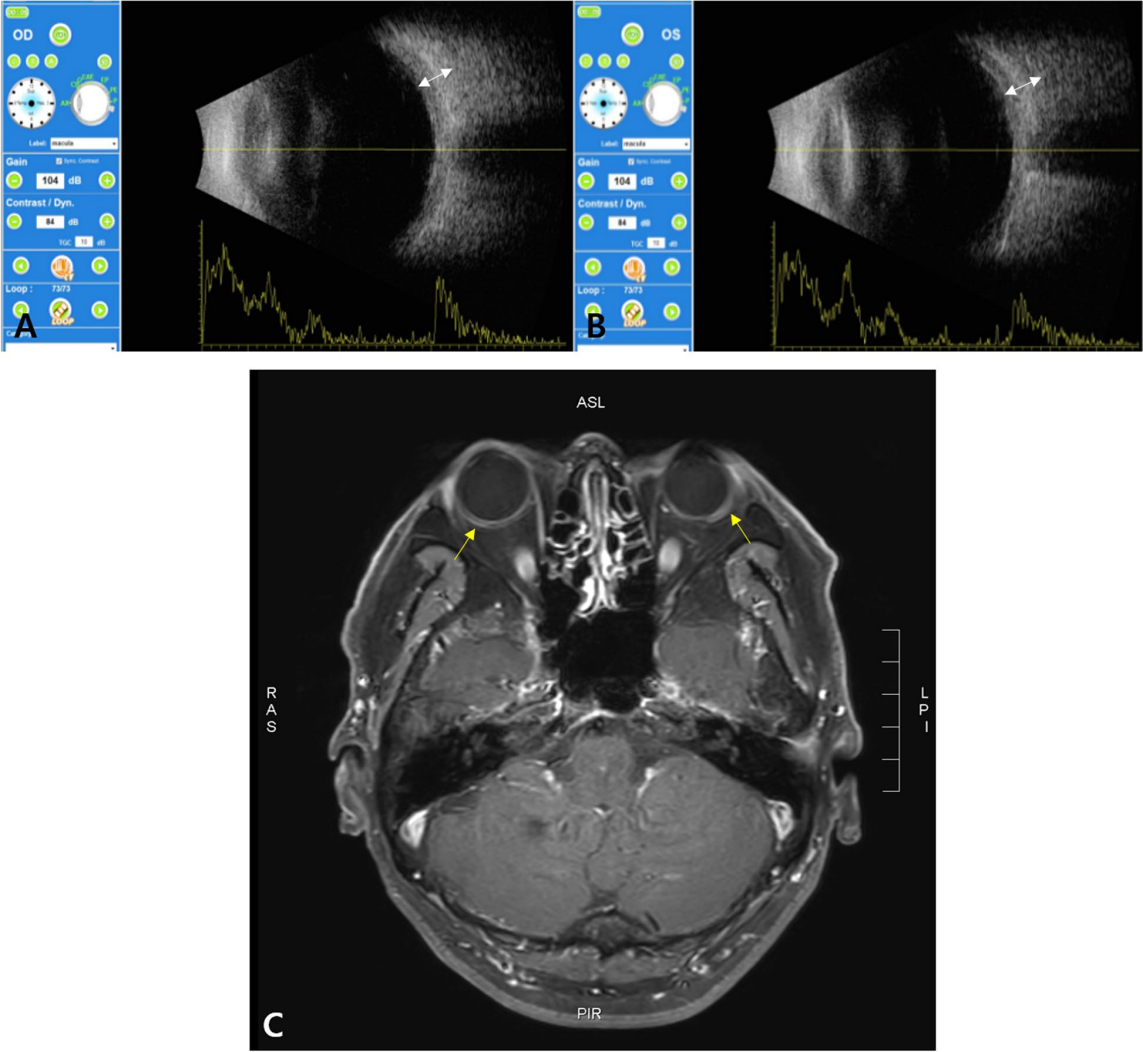
Ultrasonography and magnetic resonance imaging (MRI) of the patient at initial visit. Ultrasonography showed thickening of choroid and sclera (white left and right arrow) in the right (**a**) and left (b) eyes. MRI revealed bilateral signal enhancement (yellow arrow) of the sclera (**c**) in T1 fat suppression mode

**Fig. 3:**
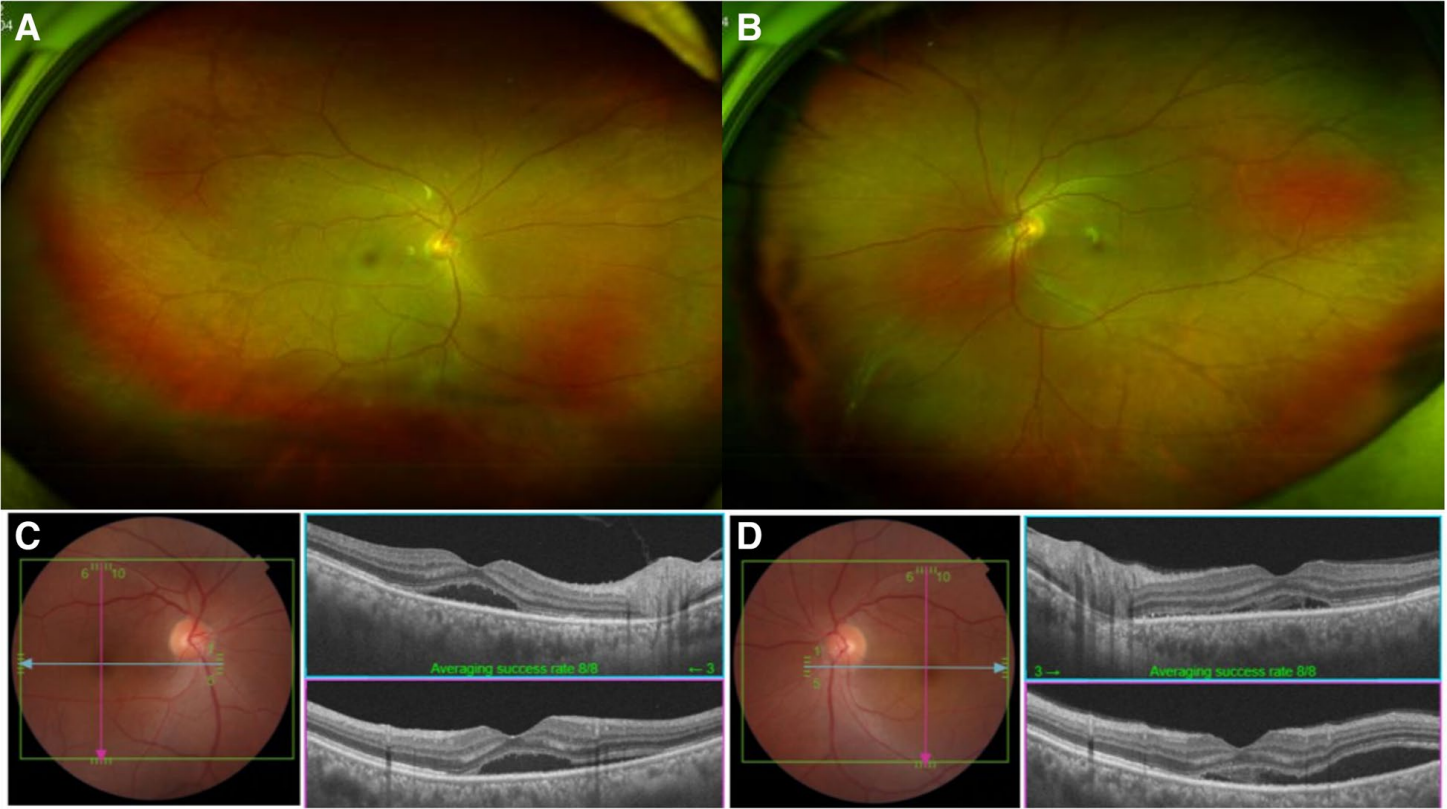
Fundus photography and optical coherence tomography (OCT) of patient after one week of high-dose steroid therapy. Fundus photography showed decreased optic disk swelling and serous retinal detachment compared with initial visit in the right (**a**) and left (**b**) eyes. OCT of the right (**c**) and left (**d**) eye revealed decreased subretinal fluid and serous retinal detachment than in the initial presentation

**Fig. 4:**
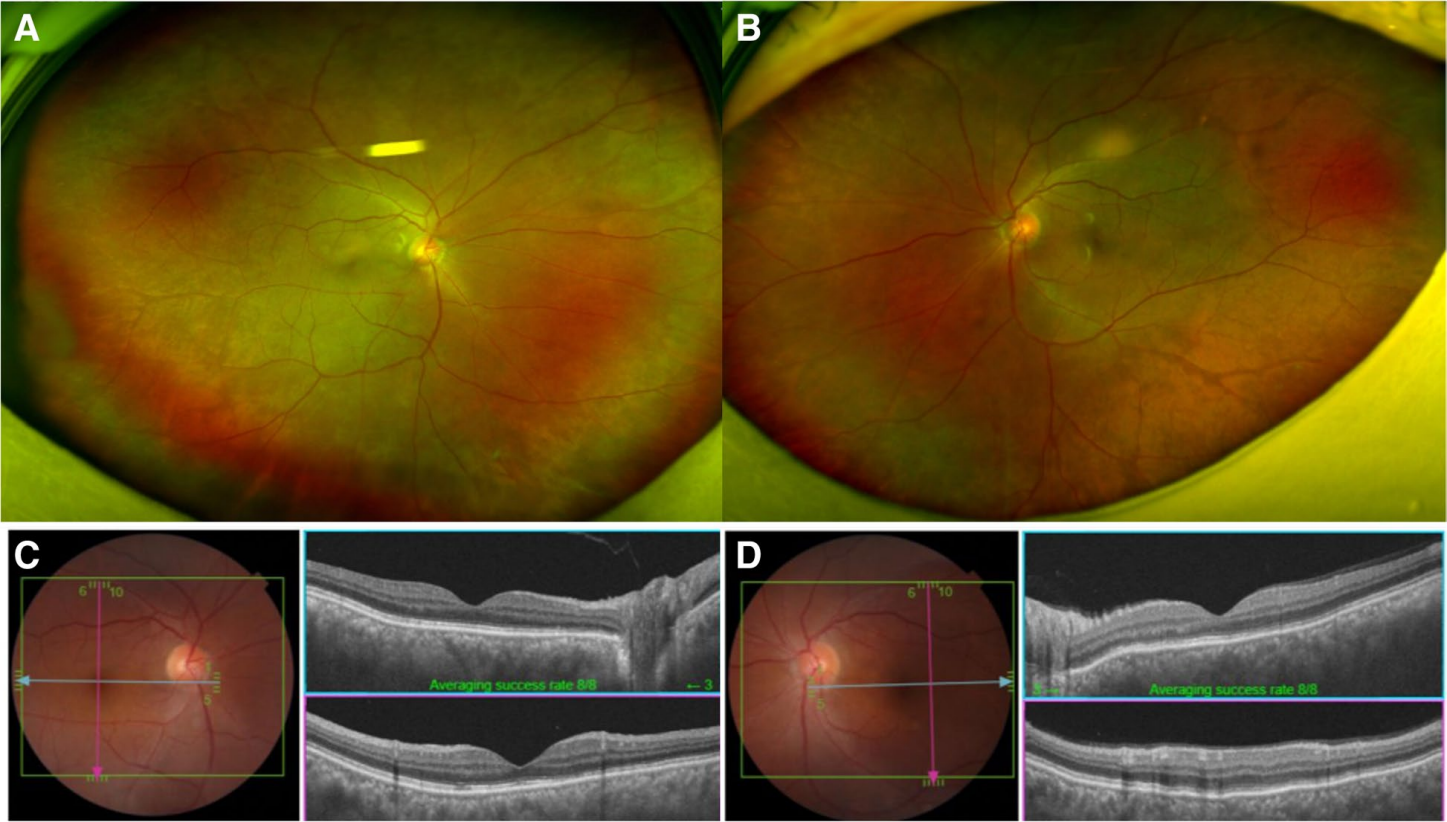
Fundus photography and optical coherence tomography (OCT) of patient after three months of high-dose steroid therapy. Fundus photography and OCT showed complete resolution of serous retinal detachment and absorption of subretinal fluid in the right (**a**, **c**) and left (**b**, **d**) eyes

**Fig. 5:**
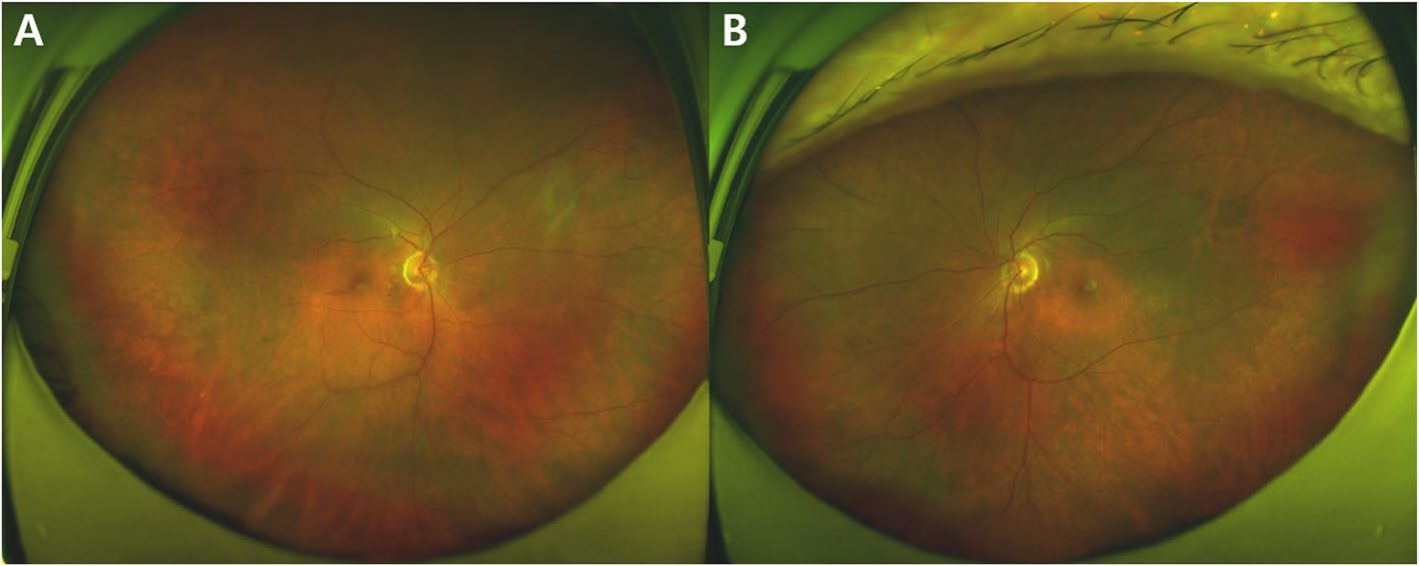
Fundus photography of patient at last visit (over a year after occurring the disease). Fundus photography showed no depigmented lesion such as sunset-glow fundus in Vogt–Koyanagi–Harada syndrome. (**a**, right ; **b**, left)

A 55-year-old male patient presented to the hospital with ocular pain and sudden bilateral vision deterioration. Phacoemulsification with intraocular lens implantation had been performed for cataract, one week before in the left eye and two weeks before in the right eye. Ophthalmic examinations before the surgery did not reveal any specific findings other than bilateral cataracts, and there were no complications during surgery. No specific family history or neurological symptoms were reported. The last measured best corrected visual acuity (BCVA) in the outpatient department after cataract surgery was 20/20 in the right eye (+ 1.00 diopter spherical equivalent) and 20/25 in the left eye (+ 0.50 diopter spherical equivalent). However, after the decrease in visual acuity in both the eyes, the BCVA was 20/100 in the right eye (+ 1.75 diopter spherical equivalent) and 20/125 in the left eye (+ 0.75 diopter spherical equivalent). The intraocular pressure measured bilaterally with a Goldmann tonometer was 11mmHg. Conjunctival and episcleral injection was shown in both eyes without ocular movement limitation in either eye. No abnormalities of the cornea and the intraocular lens, or anterior inflammation was on examination of anterior segment. Fundus examination and optical coherence tomography (SPECTRALIS® OCT, Heidelberg Engineering Inc., Heidelberg, Germany) revealed serous retinal detachment of the macula and thickened choroid (subfoveal choroidal thickness was 791µm and 794 µm in right and left, respectively) along with bilateral optic disc swelling (Fig. 1a and d). In fluorescein angiography (FA) and indocyanine green angiography (ICGA), there was no definite hyperfluorescein lesion or hot spot in the early phase. At the late phase of FA and ICGA, multiple areas of fluorescein pooling in macular area and fluorescein leakage of optic nerve, retina and choroid were observed. In addition, identified choroidal staining spots were seen in late phase of FA and ICGA, which are not shown in early phase and not associated with fluorescein pooling area (Fig. 1e and f). Ultrasound confirmed thickening of the sclera and choroid (Fig. 2a and b), and fat-suppressed T1-weighted magnetic resonance imaging (MRI) revealed bilateral thickening and signal enhancement of the sclera (Fig. 2c). No specific findings were observed in systemic examination, including chest X-rays and hematological and immunological tests. Therefore, high-dose systemic steroid treatment was planned based on the diagnosis of bilateral posterior scleritis. Methylprednisolone 1 g/day was administered intravenously, amounting to a total of 3 g in 3 days. Subsequently, oral prednisolone was given at a dose of 60 mg/day and reduced by 10 mg/day at 1-week intervals. After one week of high-dose steroid treatment, the BCVA improved to 20/25 in both eyes. Furthermore, reduced bilateral optic disc swelling and subretinal fluid of macula were observed in fundus examination and optical coherence tomography (Triton™, Topcon, Tokyo, Japan) respectively (Fig. 3). Three months after the start of treatment, the BCVA improved to 20/20 in both eyes. Moreover, in fundus examination and optical coherence tomography (Triton™, Topcon, Tokyo, Japan), serous retinal detachment disappeared bilaterally and only choroidal folds were observed (Fig. 4). The patient is being followed up, and there has been no relapse or worsening over a period of one year (Fig. 5).

## Discussion and conclusions

Posterior scleritis is a rare inflammatory disease of the sclera probably accompanied by immunologic diseases such as rheumatoid arthritis, Wegener granulomatosis, and systemic vasculitis, as well as infectious diseases such as syphilis and tuberculosis. Therefore, a thorough examination is required to rule out systemic diseases, and the condition should be differentiated from inflammatory ophthalmic diseases like Vogt–Koyanagi–Harada syndrome [1, 4].

Though the most common cause of bilateral posterior scleritis is idiopathic, there are reports that it can occur in association with tuberculosis and giant cell arteritis [5–7]. Bilateral occurrence of posterior scleritis varies in each report; however, an incidence of 15.78% was reported in a study that analyzed 18 patients with the condition [5].

The present case involved posterior scleritis in both eyes after sequential bilateral cataract surgery, which, previously, has not been reported. There have been reports of necrotizing keratitis after cataract surgery, especially in patients with rheumatoid arthritis or collagen vascular disease [8, 9]. However, in this case, autoimmune abnormalities or vasculitis were not observed in systemic and immunological tests, and neurological symptoms were also not found. In addition, anterior chamber inflammation and granulomatous keratoprecipitate were not observed, which differentiating the condition from sympathetic ophthalmia after cataract surgery.

Moreover, if a severe bilateral retinal detachment is observed, it is necessary to differentiate it from other diseases like central serous retinopathy and Vogt–Koyanagi–Harada syndrome. This syndrome spreads to the central nervous system, resulting in systemic symptoms and granulomatous inflammation of the cornea. Keratoprecipitae of the cornea and severe vitreous body inflammation may occur, along with several independent areas of serous retinal detachment that merge over time [4]. However, in this case, neurological symptoms such as tinnitus, neck stiffness, alopecia, and skin vitiligo observed in Vogt–Koyanagi–Harada syndrome were not present. Besides, anterior chamber inflammation and keratoprecipitate were not noted. Recently a cases of coronavirus disease-2019 (COVID-19) vaccination associated scleritis were reported [10]. In this report, authors postulate that posterior scleritis occurred as an immune-mediated reaction. However, in our case, patient has no abnormality in hematologic and immunologic test and has no history of COVID-19 infection or COVID-19 vaccination. Therefore, scleral inflammation of our case might be initiated by the surgical trauma of cataract surgery and the inflammation might aggravate by an immunological reaction.

In addition, T-signs often seen with ultrasound examination in posterior scleritis were not visible in this case. In a previous report of bilateral posterior scleritis, T-signs were observed in 11.11%, showing low diagnostic specificity [5]. Therefore, as in this case, additional examination using MRI is required for diagnosing posterior scleritis.

In posterior scleritis management, systemic high-dose steroid treatment has been predominantly reported, and immunosuppressive agents and non-steroidal anti-inflammatory drugs are also being used [11]. However, in bilateral posterior scleritis, oral steroid treatment alone is effective in only about 22% of the patients, and intravenous (IV) high-dose steroid treatment is often necessary to control inflammation and to prevent relapse [5]. In this case, we treated the patient with high-dose steroids, consisting of methylprednisolone 1 g/day, amounting to a total of 3 g IV administration over a period of three days, followed by oral prednisolone at a dose of 60 mg/day, with a weekly tapering of 10 mg/day. The inflammation was controlled, and did not recur.

This is the first report on bilateral posterior scleritis occurring after sequential bilateral cataract surgery. We found that rapid treatment with high-dose systemic steroids with tapered per oral steroid medication was successful in controlling inflammation and preventing relapse. Hence, if suspected, prompt diagnosis through means such as MRI and active treatment with high-dose-steroids are necessary.

## Data Availability

Data sharing is not applicable to this article as no datasets were generated or analysed during the current study.

## Abbreviations

BCVA: Best-corrected visual acuity
COVID-19: Coronavirus disease-2019
FA: Fluorescein angiography
ICGA: Indocyanine green angiography
IV: Intravenous
MRI: Magnetic resonance imaging
